# Case Report: Unusual case of candy wrapper aspiration

**DOI:** 10.12688/wellcomeopenres.22993.1

**Published:** 2024-09-16

**Authors:** Santosh Adhikari, Amod Rayamajhi, Tribhuwan Bhattarai, Shristi Upadhyay, Sanjeet Kumar Shrestha, Buddha Basnyat, Ajit Rayamajhi

**Affiliations:** 1 Department of Pediatric Medicine, Kanti Children's Hospital, Kathmandu, Bagmati, 44600, Nepal; 2 Global Health Research and Medical Intervention for Development, Kanti Children’s Hospital, Kathmandu, Bagmati, 44600, Nepal; 3 Oxford University Clinical Research Unit Nepal, Kathmandu, Bagmati, 44600, Nepal; 4 Department of Pediatrics, National Academy of Medical Sciences, Kanti Children's Hospital, Kathmandu, Bagmati, 44600, Nepal

**Keywords:** foreign body aspiration, flexible bronchoscopy, candy wrapper, Nepal

## Abstract

Foreign body (FB) aspiration is a life-threatening medical emergency that usually presents with a history of choking episodes, followed by cough and shortness of breath. However, when the signs and symptoms are subtle, they can be easily missed by the parents or the child, causing delays in the diagnosis and management, suspecting other respiratory illnesses. Here, we report an eight years old neurologically stable girl without a history of choking episodes, with only subtle respiratory symptoms, with a candy wrapper stuck in the left bronchus missed by X-ray and computer tomography (CT)- scan of the chest and diagnosed and removed by flexible bronchoscopy.

## Plain language summary

The patient was admitted, managed and discharged after ten days from Pediatric Ward of Kanti Children’s hospital, Kathmandu, Nepal in January of 2024. Foreign body (FB) aspiration is a life-threatening medical emergency usually presenting with history of choking episode followed by repeated episodes of cough and shortness of breath. It usually occurs in children below 3 years of age. It is in older children and the risk of FB aspiration occurs if they are not fully conscious. Chest x-ray, CT scan of chest and rigid bronchoscopy are the investigations performed for the diagnosis of FB aspiration which usually occurs in the airway of the right side of the lung. We report an unusual case of candy wrapper aspiration in left bronchus in an 8-years-old girl who was fully conscious, without any choking episodes, with two subtle episodes of cough and breathing difficulty, which was diagnosed and removed by flexible bronchoscopy. Her x-ray and CT scan of chest were inconclusive. She was being treated as a case of bacterial pneumonia and was suspected and investigated for tuberculosis of the lungs because of prolonged duration of illness of one month. Hence, we suggest all children, including older ones who are fully conscious, with recurrent or prolonged cough or breathing difficulty, to be suspected of FB aspiration, even in the absence of choking episode, typical symptoms and signs of aspiration and atypical x-ray or CT scan of chest, which could be lifesaving.

## Introduction

Foreign body (FB) aspiration and lodgment in the airway are life-threatening medical emergencies in children, especially in toddlers. Seeds, peanuts, food particles and toys are the most common objects aspirated by toddlers, whereas coins, paper clips, pins, and pen caps are by older children ^1^. Older children are reported to develop neurological dysfunctions, psychological problems and alcohol or substance abuse after accidental aspirations ^2^.

During the early stage, after aspiration of the organic FB, mucosal inflammation and edema of the respiratory tract occur. Later on, granulation tissue may form, causing vague symptoms of cough, shortness of breath, and wheeze, mimicking bronchial asthma. They may also be considered to have recurrent/persistent pneumonia ^3^.

Rigid bronchoscopy is the most sensitive and specific tool for the diagnosis and treatment of FB. Failure of parents to notice and mention choking events, the absence of characteristic symptoms in children, and misleading radiological findings make the diagnosis of FB aspiration difficult. Since X-ray imaging to detect FB is based on the radio-dense property of the foreign body, translucent foreign bodies may not be detected on plain chest radiographs ^4^.

We report an 8-years-old child with FB aspiration in the left bronchus who was initially suspected and treated for many respiratory illnesses, including pulmonary tuberculosis.

## Case report

An eight-year-old girl from Dhading, a neighboring district of Kathmandu, presented to Kanti Children’s Hospital in Kathmandu, Nepal with complaints of cough for 3 days, which was insidious in onset and gradually progressive, productive in nature with yellowish sputum associated with tightness and pain in the chest during coughing along with progressive difficulty in breathing. There was also a history of intermittent fever of up to 102 ^0^F after the onset of cough. She had no history of dizziness, altered sensorium, seizures, or cyanosis. The patient had a history of fever, cough, and shortness of breath one month ago which subsided after treatment with amoxicillin and clavulanic acid for 7 days. She was the second child of a nonconsanguineous marriage. There was no history of any chronic illness or tuberculosis in any family member. She was a Grade 3 student with average academic performance and had received all immunizations according to the national immunization schedule.

On arrival at the emergency department, she had tachypnea (respiratory rate, 36 breaths/min), normal SpO2 of 96% at room air, tachycardia (pulse rate 110/ minute), fever (temperature of 100.8°F), normal blood pressure (100/60 mmHg in the right arm), and normal capillary refill time (< 2 seconds). On chest auscultation, air entry was significantly reduced on the entire left side of the chest, heart sounds were normal and there were no murmurs. Abdominal and nervous system examination results were normal.

**Figure 1.**
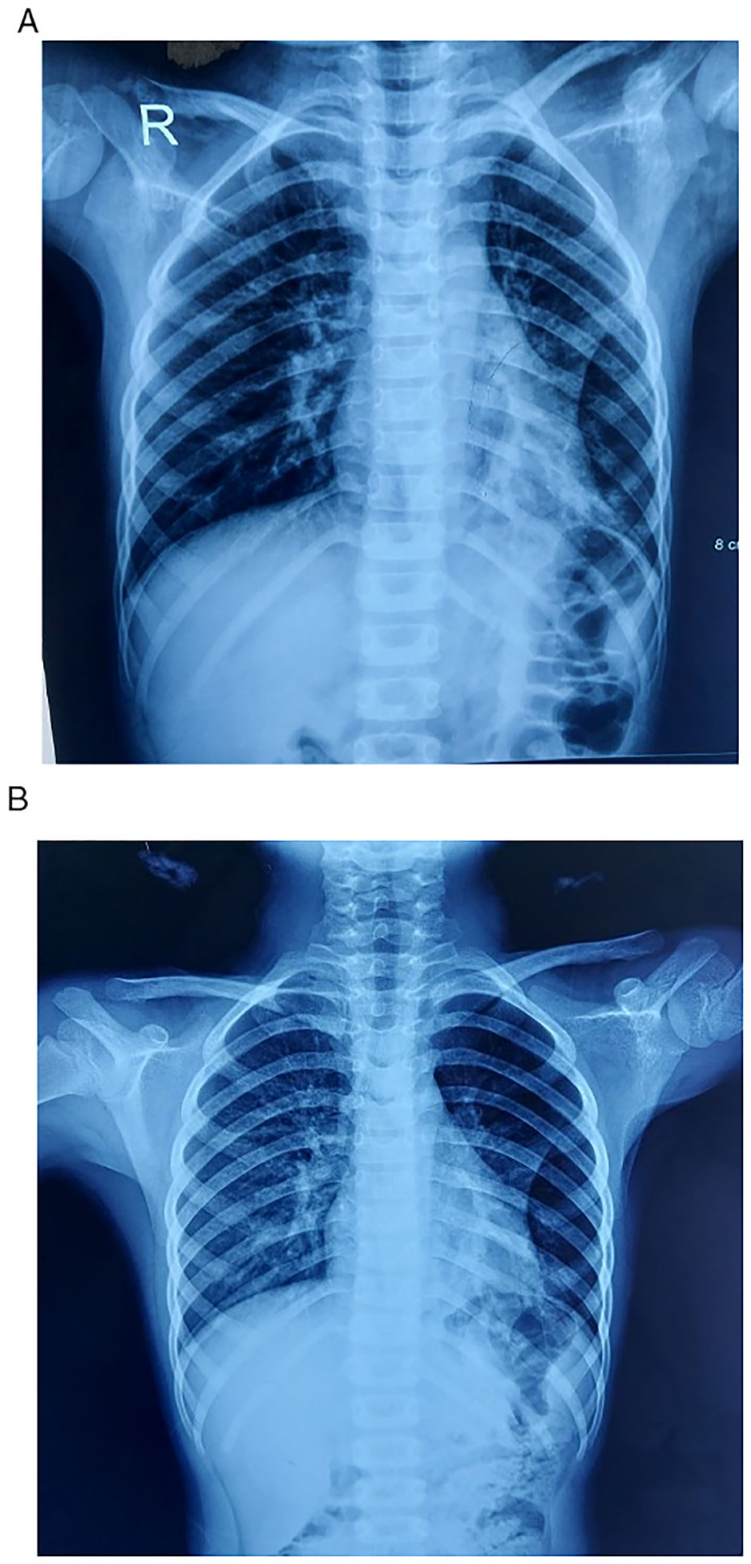
**A**: X-ray of chest on the day of admission showed wedge shaped homogenous opacity with loss of silhouette with heart border which suggested sub-segmental collapse with air trapping. **B**: Repeat x-ray of chest on 5 ^th^ day of admission showed the opacity to be reduced but no change in sub-segmental collapse. **A** & **B**: There were no significant differences in radiological findings after 5 days of antibiotic treatment between the two x-rays.

**Figure 2.**
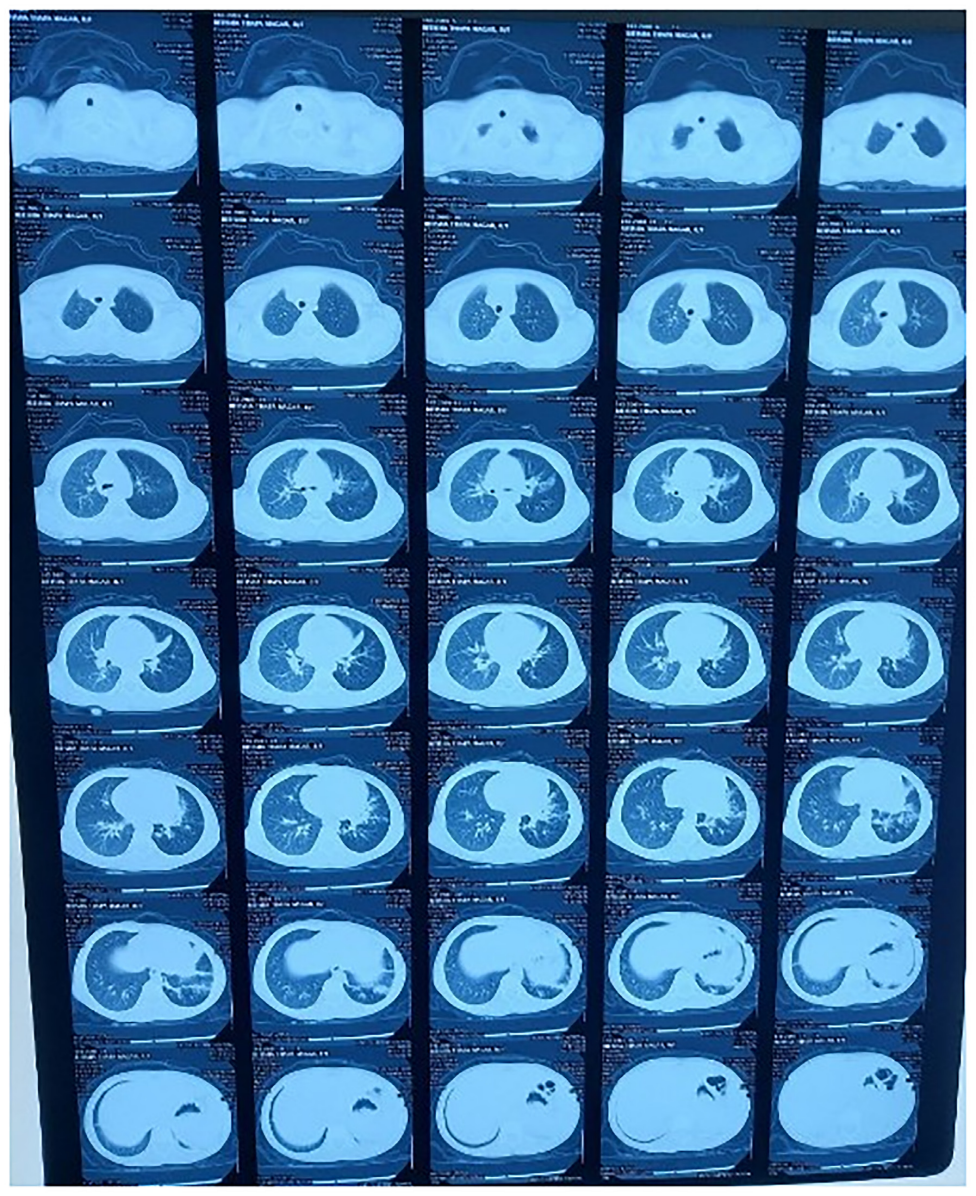
CT scan of the chest done on sixth day of admission. Shows collapse consolidation of superior lingular segment of left upper lobe and multiple centrilobular nodules with tree-in-bud appearance consolidation.

**Figure 3.**
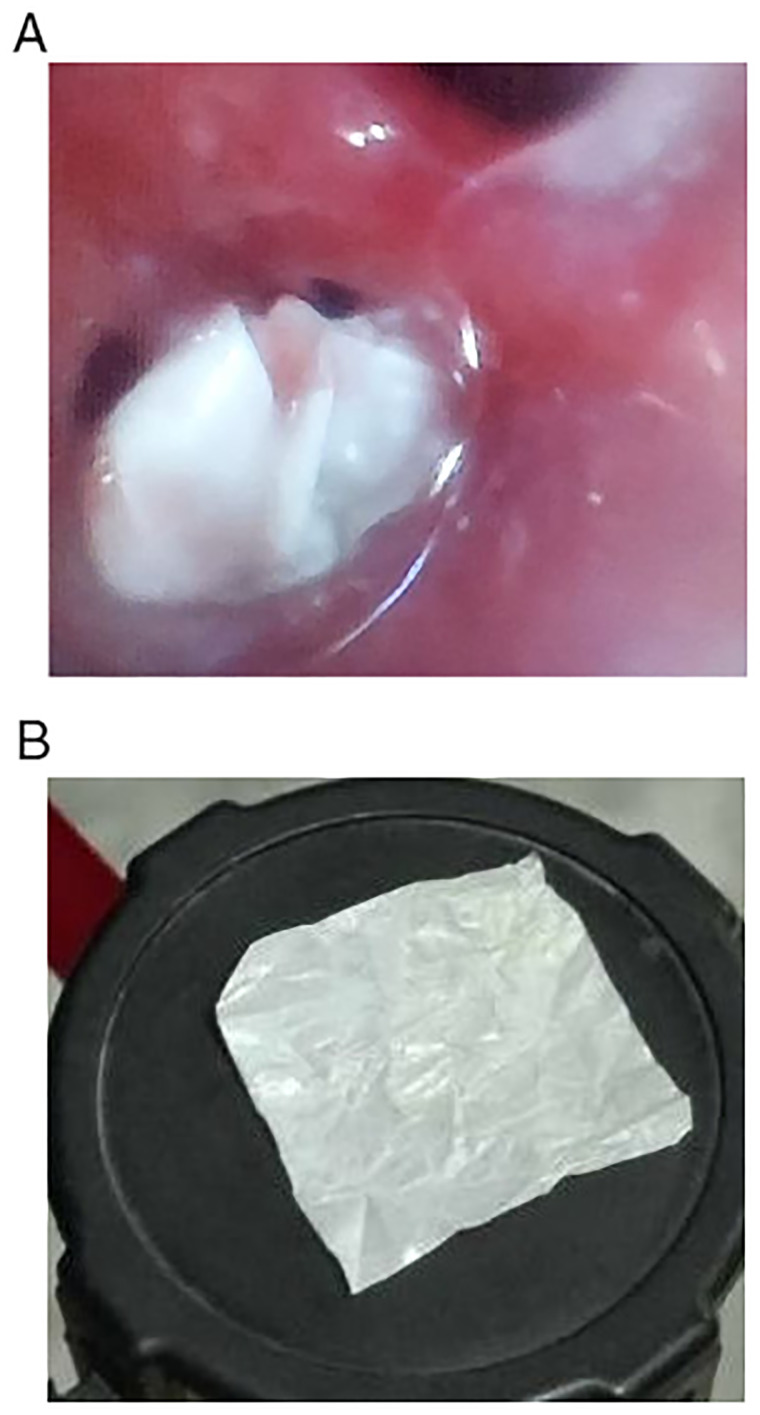
**A**: Shows white plastic candy wrapper in left bronchus. **B**: Shows white plastic candy wrapper after removal. **A** & **B**: White candy wrapper as seen in the bronchus (Figure 3 **A**) and after removal (Figure 3 **B**).

On investigation, hemoglobin was 12.4 gm/dl, total leukocyte counts 19,500/ mm ^3^ (neutrophil 90%, Lymphocytes 10%), platelet count 376,000/mm ^3^, C-reactive protein 6 mg/dl and erythrocyte sedimentation rate 25 mm/hour. Tubercular skin test result was negative (0 mm in 72 hours), and sputum for gene x-pert did not reveal Mycobacterium tuberculosis. Her renal and liver functions were normal. The child was admitted to the pediatric ward and received intravenous Ceftriaxone and Flucloxcillin for 10 days. Although she became afebrile and her fast breathing improved, repeat chest radiography done on 5 ^th^ day of admission showed no improvement in the initial radiological findings ( Figures 1A and 1B). Furthermore, a computer tomography (CT) scan of the chest was performed on the 6 ^th^ day of admission, which revealed air trapping with hyperinflation of the left lung field, collapse consolidation of the superior lingular segment of the left upper lobe, and multiple centrilobular nodules with tree-in-bud appearance consolidation and bronchial wall thickening in the left lower lobe ( Figure 2). Three days after the chest CT scan, flexible bronchoscopy (Fujifilm Company, India) was performed under general anesthesia, revealing a 4 x 3 centimeter ^2^ white plastic candy wrapper. Figure 3A shows the wrapper in the left bronchus and Figure 3B shows the same wrapper after removal from the bronchi.

## Discussion

Most cases of FB aspiration initially present with choking, cough, and respiratory distress, which may be followed by fever, stridor, chest pain, and sternal discomfort over time. The site of impaction, degree of obstruction, and degree of mobility of the FB in the respiratory tract determine the severity of the clinical symptoms and signs. However, occasionally, because of the inability of parents to witness the actual event, lack of suspicion by the treating physician because of subtle presenting symptoms, similarity of symptoms of acute exacerbation of bronchial asthma, or severe pneumonia may lead to delayed diagnosis of FB in the airway ^5^.

Three clinical phases have been reported in cases where there has been a delay in recognition of the presence of FB in the airway. In the initial phase, the patient reported choking, gagging, and paroxysms of cough. Then, there is an asymptomatic second latent phase, where coughing and protective mechanisms become fatigued. This is followed by a third phase, where all complications such as obstruction, erosion, or infections occur, causing pneumonia, atelectasis, abscess, or granulation tissue formation, leading to narrowing of the airway lumen ^6^.

In our case, the incidence of cough and fever which occurred one month before presentation could have been the actual event of choking and FB aspiration, although the parents and the child themselves were unsure of the event. Subsequently, the child was asymptomatic for a month, when she again developed symptoms that had brought her to the hospital. This progression of events is typical of unnoticed FB aspirations. A similar episode of unnoticed FB aspiration has also been reported in the past, where an eight-year-old child required multiple hospital admissions for repeated episodes of cough and dyspnea, which temporarily improved after medical treatment for up to 15 months. The aspirated FB had also been initially missed by rigid bronchoscopy but later diagnosed by flexible bronchoscopy examination ^7^. Another similar incidence has also been reported where an eleven years old boy had received treatment, on and off with bronchodilator for 18 months suspecting bronchial asthma, which was later confirmed to be FB stuck in the bronchus ^8^. Radiolucent FB, such as flowers or plastic whistles, may not completely occlude the airway or cause choking sensation or breathlessness and could easily be missed by radiography of the chest and mislead physicians to not suspect FB aspiration.

Surprisingly, in our case, even the age of the neurologically well child did not favor FB aspiration since accidental FB aspiration is more common in healthy toddlers. However, there have been several reports of FB aspiration in neurologically well child above five years of age ^7, 8^. Similarly, in our case, the FB was retrieved from the left main bronchus, whereas the right bronchus was reported to be the most common site for dislodgement. This could have been possible because of its wider caliber and vertical course ^9^. It may also occur in children younger than 15 years of age in whom the difference in the left and right bronchi is less pronounced because of the immature airway and symmetric tracheal take-off angle ^10^.

Although features of hyperinflation, atelectasis, collapse and mediastinal shift should arouse suspicion of FB in Chest x-ray with a sensitivity up-to 70%, it has also been reported to appear normal in a significant proportion of cases ^11^. Computed tomography (CT) scan of chest is more accurate investigation than chest x-ray for the diagnosis of FB aspiration ^12^. CT scan of chest, in our case, was inconclusive, probably because of excessive granulation tissue and secondary infective pathology because of prolonged duration of aspiration.

Rigid bronchoscopy is considered the gold standard method for the diagnosis of FB dislodged in the central airway ^13^. However, flexible bronchoscopy along with fluoroscopy in expert hands is proving to be more effective for the diagnosis and removal of FB ^14^. More importantly, if it is located in the lobar bronchus with more distal displacement, rigid bronchoscopy would not be helpful at all; thus, the only option would be flexible bronchoscopy. There are also reports in the past where FB have been missed by rigid bronchoscopy but have been detected by flexible bronchoscopy ^7^. Similarly, in our case, the FB was diagnosed and removed by flexible bronchoscopy.

## Conclusion

Choking episodes may not be observed in all of the children with FB aspiration. They may not always have typical symptoms or signs of aspiration. FB aspiration must be suspected in all children who have recurrent or prolonged respiratory symptoms, even though chest radiography and CT scans may not be suggestive. Flexible bronchoscopy is a useful tool for diagnosing and removing FB from the distal bronchus.

## Consent

Written and verbal informed consent for publication of their clinical details and clinical images was obtained from the parents of the patient.

## Data Availability

No data are associated with this article.
