# Supplementary material for: Case Report: Unusual case of candy wrapper aspiration
Source: Wellcome Open Res. 2024 Sep 16;9:525. [Version 1] doi: 10.12688/wellcomeopenres.22993.1 (PMC11499866; doi:10.12688/wellcomeopenres.22993.1)
Supplement: Peer review history

# Reviewer report: Reviewer response for version 1

**DOI:** 10.21956/wellcomeopenres.25319.r103754

**Published:** 2024-10-28

**Authors:** Evaristus Afiadigwe

**Affiliations:** 1 Department of Otorhinolaryngology, Nnamdi Azikiwe University, Nnewi, Nigeria

This is a fascinating case report. Interestingly, even with negative radiological findings, the Clinician did not give up. However, I have the following observations:

 1. The statement "Older children are reported to develop neurological dysfunctions, psychological problems and alcohol or substance abuse after accidental aspirations ^"   ^is not correct. Rather such neurological disorders predispose the older children and adults to foreign body aspiration. 

 2. History is very important in foreign body management whether in the airway or gastrointestinal system. therefore if an 8-year-old child could not give a history of a foreign body and no caregiver also did, it has to be stated clearly as an important negative. From my experience, if the index patient is not mentally deranged in any way, fear may have caused her to withhold the history. 

 3. As already stated, this patient presented at the latent phase of aspiration and hence the symptoms were already suppressed. So it should be stated in the second paragraph of the discussion 

 4. Apart from the reasons adduced in paragraph 4 of the discussion for the left bronchial site of the foreign body, it has been documented that non-occlusive foreign bodies could migrate in the course of breathing and coughing. It is more likely for foreign bodies to migrate from the larger and more vertical right bronchus to the left than the reverse.

 In general this is a good paper and I recommend acceptance with these minor corrections.

Are enough details provided of any physical examination and diagnostic tests, treatment given and outcomes?

Yes

Is the case presented with sufficient detail to be useful for other practitioners?

Partly

Is sufficient discussion included of the importance of the findings and their relevance to future understanding of disease processes, diagnosis or treatment?

Yes

Is the background of the case’s history and progression described in sufficient detail?

Yes

Reviewer Expertise:

Otorhinolaryngology, Head and Neck Surgery with interest in Paediatric airway

I confirm that I have read this submission and believe that I have an appropriate level of expertise to confirm that it is of an acceptable scientific standard.

# Reviewer report: Reviewer response for version 1

**DOI:** 10.21956/wellcomeopenres.25319.r103753

**Published:** 2024-10-23

**Authors:** Alaa Gaafar

**Affiliations:** 1 Department of Otolaryngology, Alexandria University, Alexandria, Alexandria Governorate, Egypt

Thank you for sending this manuscript for assessment. FB aspiration is a serious health problem in young age with possible mortality in some cases. In children most of the aspirated foreign bodies are organic in nature that is why they difficult to be visualized by X ray or CT. 

 The paper is well written and fulfilling all the aspects of a Case report presentation. I have few comments to the authors:

 1. Witnessed history from the parents or care giver of choking during eating or playing is very helpful in suspicious of FB aspiration. In this case, she is 8 years old. How did she not mention that she had experienced an attack of chocking??

 2. Any child with repeated history of respiratory tract infection and unilateral chest X ray findings is highly suspicious of neglected FB aspiration. CT scan here has no role. Actually, it caused more exposure to irradiation without any new findings rather than hyperinflation of the left lung and consolidation which were diagnosed already by X-ray.

 3. I agree that flexible bronchoscopy could be used in FB extraction especially in small, soft foreign bodies not causing central severe airway obstruction. Otherwise, Rigid bronchoscopy is the gold standard modality for extraction of foreign bodies from the airway.

Are enough details provided of any physical examination and diagnostic tests, treatment given and outcomes?

Partly

Is the case presented with sufficient detail to be useful for other practitioners?

Yes

Is sufficient discussion included of the importance of the findings and their relevance to future understanding of disease processes, diagnosis or treatment?

Yes

Is the background of the case’s history and progression described in sufficient detail?

Yes

Reviewer Expertise:

Airway surgery in adults and pediatrics

I confirm that I have read this submission and believe that I have an appropriate level of expertise to confirm that it is of an acceptable scientific standard.

# Reviewer report: Reviewer response for version 1

**DOI:** 10.21956/wellcomeopenres.25319.r99611

**Published:** 2024-10-03

**Authors:** Hüseyin Fatih Sezer

**Affiliations:** 1 Department of Thoracic Surgery, Kocaeli University Faculty of Medicine, Kocaeli, Turkey

Dear Author;

 First of all, I would like to thank you for the effort you have put into preparing the article. I have some criticisms about the article, they are presented below.

 - The purpose should be stated more broadly and clearly in the introduction section.

 - Too much disease history is given in the case section, it should be summarized in a few sentences.

 - A few special CT slices that are specially selected should be given in Figure 2.

 - The sentence shown with references 7 and 8 in the discussion is very ambitious. It should also not be forgotten that a fiber bronchoscope can be passed through the rigid bronchoscope to remove distal foreign bodies while general anesthesia is already given.

Are enough details provided of any physical examination and diagnostic tests, treatment given and outcomes?

Yes

Is the case presented with sufficient detail to be useful for other practitioners?

Yes

Is sufficient discussion included of the importance of the findings and their relevance to future understanding of disease processes, diagnosis or treatment?

Yes

Is the background of the case’s history and progression described in sufficient detail?

Yes

Reviewer Expertise:

NA

I confirm that I have read this submission and believe that I have an appropriate level of expertise to confirm that it is of an acceptable scientific standard.
